# An advanced empirical model for quantifying the impact of heat and climate change on human physical work capacity

**DOI:** 10.1007/s00484-021-02105-0

**Published:** 2021-03-05

**Authors:** Josh Foster, James W. Smallcombe, Simon Hodder, Ollie Jay, Andreas D. Flouris, Lars Nybo, George Havenith

**Affiliations:** 1 Environmental Ergonomics Research Centre, School of Design and Creative Arts Loughborough University, Loughborough, Leicestershire, LE11 3TU UK; 2 Thermal Ergonomics Laboratory, Faculty of Medicine and Health, The University of Sydney, Sydney, NSW Australia; 3 FAME Laboratory, University of Thessaly, Trikala, Greece; 4 Department of Nutrition, Exercise and Sports, University of Copenhagen, Copenhagen, Denmark

## Abstract

Occupational heat stress directly hampers physical work capacity (PWC), with large economic consequences for industries and regions vulnerable to global warming. Accurately quantifying PWC is essential for forecasting impacts of different climate change scenarios, but the current state of knowledge is limited, leading to potential underestimations in mild heat, and overestimations in extreme heat. We therefore developed advanced empirical equations for PWC based on 338 work sessions in climatic chambers (low air movement, no solar radiation) spanning mild to extreme heat stress. Equations for PWC are available based on air temperature and humidity, for a suite of heat stress assessment metrics, and mean skin temperature. Our models are highly sensitive to mild heat and to our knowledge are the first to include empirical data across the full range of warm and hot environments possible with future climate change across the world. Using wet bulb globe temperature (WBGT) as an example, we noted 10% reductions in PWC at mild heat stress (WBGT = 18°C) and reductions of 78% in the most extreme conditions (WBGT = 40°C). Of the different heat stress indices available, the heat index was the best predictor of group level PWC (*R*^2^ = 0.96) but can only be applied in shaded conditions. The skin temperature, but not internal/core temperature, was a strong predictor of PWC (*R*^2^ = 0.88), thermal sensation (*R*^2^ = 0.84), and thermal comfort (*R*^2^ = 0.73). The models presented apply to occupational workloads and can be used in climate projection models to predict economic and social consequences of climate change.

**Supplementary Information:**

The online version contains supplementary material available at 10.1007/s00484-021-02105-0.

## Introduction

Human exposure to increased environmental heat directly impacts the global economy by decreasing occupational productivity during work hours (Flouris et al. 2018; Hsiang et al. 2017; Kjellstrom et al. 2018). The impact of hot weather on worker productivity was estimated to cost Australia EUR 5.52 billion per year (Zander et al. 2015), and in Germany, heat-related productivity losses in 2004 were projected to cost between EUR 686.64 million and EUR 3.02 billion (Hübler et al. 2008). Occupational heat stress already has daily negative health and productivity impacts in many parts of the world and is therefore not precipitated by heat waves per se (Flouris et al. 2018; Kjellstrom et al. 2018). To correctly quantify the impact of environmental heat (mild to extreme) on human physical work capacity (PWC), accurate equations are required, which relate PWC to a wide range of thermal conditions. We describe below why existing models (Dunne et al. 2013; Kjellstrom et al. 2018; Zivin and Neidell 2014) presently used to inform those predictions have limited applicability, especially for use on a global scale. These considerations justify the development of a new series of empirically derived equations intended to quantify the loss in PWC more precisely across a wide range of environmental conditions. Such empirical equations have immediate application for those striving to evaluate the productivity and, thus, economic consequences of hot weather, particularly in different climate change scenarios arising from variations in projected greenhouse gas emissions.

## Previous models: a critical analysis

Efforts to model the loss in PWC with increasing heat have taken three distinct forms (Dunne et al. 2013; Kjellstrom et al. 2018; Zivin and Neidell 2014). In the first, with an absence of empirical data, Dunne et al. (2013) generated functions based on the American Conference of Governmental Industrial Hygienists (ACGIH)–recommended work/rest ratios for a given wet bulb globe temperature (WBGT), designed to limit core temperature from exceeding 38.0°C. Those functions are highly conservative because their objective is to minimise the risk of core body temperature of the average worker exceeding 38.0°C (which would prevent workers at the top end of the body temperature distribution to exceed 41°C with risk of heat illness) (Malchaire et al. 2001). Second, the ACGIH recommendations were never intended to represent the decline in labour output and are unlikely to accurately predict output from self-paced work in a large and diverse working population. Thus, while ACGIH recommendations show that continuous work is *safe* at 26°C WBGT, and therefore indicate no productivity loss in Dunne et al.’s (2013) model, physiological studies indicate that work output is reduced in this scenario, due to elevations in cardiovascular strain (Galloway and Maughan 1997).

Second, Kjellstrom et al. (2018) modelled field data from agriculture (Sahu et al. 2013) and gold mining (Wyndham 1969) and demonstrate a nonlinear (sigmoidal) decrease in PWC as a function of WBGT. Those data represent an improvement in ecological validity because the source data are based on scenarios where workers could freely adjust their pace. However, the source data (Sahu et al. 2013; Wyndham 1969) are highly context specific and, as such, are of limited utility on a global scale. Firstly, the reference condition to which subsequent hot trials were compared was already warm, i.e. 27°C WBGT, which far exceeds optimal thermal conditions for human performance and thus greatly affects the sensitivity of the model at modest levels of heat. Secondly, the model is based only on well-conditioned, highly heat acclimatised, and incentivised workers, which represents the upper limit of PWC in relation to each climate, but will over-predict PWC in less fit, unacclimatised workers. The latter is relevant for those who reside in climates that experience more transient or unaccustomed peaks in temperature for which they are not physically prepared. To quote Cyril Wyndham, the primary source for the data used by Kjellstrom et al. (2018), “*these curves apply only to men in the high state of acclimatization of the Bantu in the gold mines in South Africa and to men carrying out physical work at a moderate rate under direct supervision*” (Wyndham 1969). Finally, the PWC predictions from Dunne et al. (2013) and Kjellstrom et al. (2018) can only be adjusted based on WBGT, which limits their utility if an alternative heat stress metric (i.e. universal thermal climate index (UTCI), wet bulb, heat index, humidex) is preferred. A global model should have the capacity to be adjusted to incorporate any heat stress assessment metric, of which many are currently adopted for different applications (Havenith and Fiala 2015).

Finally, Zivin and Neidell (2014) model the impact of hot weather on the decrease in time allocated to labour per day, based on data from the American Time Use Survey (ATUS) (Hamermesh et al. 2005) combined with local weather station data. Hsiang et al. (2017) use the model to evaluate the contribution of labour loss to economic impacts of climate change in the USA. For industries considered vulnerable to heat, the model demonstrates reductions of up to 1-h lost labour time per day time when air temperature reaches 38°C. Importantly however, the model does not address the heat-induced reduction in labour effort *during* work (presenteeism) and is apparently insensitive to any change in humidity, a key determinant of the overall heat stress intensity (Malchaire et al. 2001; Maughan et al. 2012; Raymond et al. 2020). Field studies demonstrate that functional/quality labour time is very sensitive to heat, with total output *during* work declining with increasing heat (Ioannou et al. 2017; Kalkowsky and Kampmann 2006; Sahu et al. 2013). Furthermore, presenteeism formed a major contribution to heat-induced reductions in economic output in Australia (Zander et al. 2015).

The aim of this study was to produce an advanced, novel, empirical model for the loss in PWC with environmental heat stress. We aim to generate models of PWC based off a suite of heat stress assessment metrics, air temperature and humidity, and body temperatures. We intend the models to be used primarily for projecting the impact of heat waves and climate change on human physical work capacity.

## Materials and Methods

### Location and timeline

The data collection took place in custom-made environmental chambers located within the Environmental Ergonomics Research Centre, Loughborough University. Data collection began in July 2017 and terminated in October 2019.

### Participants

Young adult males, primarily from a student population, were recruited for this study. The total number of trials completed by each participant (*n*=40) varied (average = 12, range = 4 to 35). Participant characteristics are shown for each experimental group in Table 1.

**Table 1:** Participant characteristics. Data are presented as means ± standard deviation. The data range is presented in parentheses.

Variable	Low-clothing coverage (*n* = 20)		High-clothing coverage (*n* = 20)	
Age (years)	25 ± 3	(20–29)	24 ± 2	(20–28)
Height (cm)	*178 ± 5*	(170–190)	180 ± 6	(172–192)
Mass (kg)	77 ± 11	(61–101)	79 ± 11	(62–99)
Body surface area (m^−2^)	1.9 ± 0.2	(1.7–2.3)	2.0 ± 0.2	(1.7–2.3)
Body mass index (kg∙m^−2^)	24 ± 2	(21–29)	24 ± 2	(21–29)
Body fat (%)	18 ± 5	(11–26)	15 ± 5	(8–26)
*V̇*O_2max_ (L·min^−1^)	3.8 ± 0.8	(2.7–6.2)	4.0 ± 0.7	(2.7–5.3)
*V̇*O_2max_ (mL·kg^−1^·min^−1^)	50 ± 9	(40–67)	51 ± 8	(39–64)

*V̇O*_*2max*_ maximal oxygen consumption, *L* litres, *mL* millilitres

### A fixed cardiovascular strain protocol to model self-pacing

In the present study, we define PWC as *‘the maximum physical work output that can be reasonably expected from an individual performing moderate to heavy work over an entire shift’*. To more effectively investigate how heat stress impacts PWC, we designed a laboratory protocol to simulate these pacing behaviours, capturing the reduction in performance in warming climates, relative to a cool reference climate of 15°C. This air temperature was chosen to limit any effect of temperature on heart rate without causing substantial cold stress. Air temperatures as low as 20°C have been shown to decrease PWC relative to a cooler climate (Galloway and Maughan 1997), and 15°C has been used previously to determine the impact of heat stress on human physical performance (Marino et al. 2000). The protocol aimed at measuring the amount of work the body can generate at a fixed, maximally acceptable cardiovascular strain (130 b∙min^−1^) across a broad spectrum of air temperature (*T*_a_, 25–50°C) and relative humidity (20–80%). The spectrum of environmental conditions represents mild exposures to more extreme levels that extend into future worst-case greenhouse gas emission scenarios (i.e. WBGT 18–40°C) (Kjellstrom et al. 2018; Pal and Eltahir 2016). The experiments were up to 1-h duration and took place within an environmental chamber, where participants walked on a treadmill that automatically adjusted its speed and incline to maintain a stable heart rate of 130 b·min^−1^. A large body of evidence from the field demonstrates that workers pace themselves based on their heart rate/perceived exertion, resulting in similar values for working heart rate independent of the climate (Kalkowsky and Kampmann 2006; Mairiaux and Malchaire 1985; Miller et al. 2011; Vogt et al. 1983; Wyndham 1969), but at the cost of productivity (Kjellstrom et al. 2018). Recently, this approach was employed successfully to investigate the effect of electric fans on work output in mild heat (Jay et al. 2019).

The fixed cardiovascular strain protocol is highly sensitive to changes in PWC with heat because as the environmental heat load increases, a growing proportion of the 130 b·min^−1^ heart rate is used to ensure delivery of warm blood to the skin for heat loss (Rowell 1974). Consequently, a lower proportion of the cardiac output (blood leaving the heart per minute) can be used to fuel muscular work during heat stress for a fixed heart rate, resulting in a reduced total work output (Rowell 1974). The fixed heart rate protocol likely minimises any impact of the environment on stroke volume, since any reduction in stroke volume during heat stress is related to the increase in heart rate (due to impacts on ventricular filling time) (Chou et al. 2019). The target working heart rate was chosen based on three key sources. Firstly, the World Health Organisation classification of relative work intensities indicates that 130 b·min^−1^ during the work periods represents the demarcation between moderate and heavy strain (Andersen 1978), considered to be the maximal acceptable workload for sustained work periods (Bernard and Kenney 1994). Secondly, field observations indicated that physical work in the heat was regulated at a working heart rate close to 130 b·min^−1^ in miners (Wyndham 1973) and in glass furnace workers (Mairiaux and Malchaire 1985), regardless of the heat load of the environment. Thirdly, during a working period of four work bouts with different heat loads, absolute heart rate was on average 130 b·min^−1^ at the end of each self-paced work bout (Vogt et al. 1983). The field data indicate that 130 b·min^−1^ is an acceptable upper level of physiological strain for the maintenance of physical work in thermally challenging environments, at the cost of reduced muscular work as the heat stress increases.

### Limiting the development of physiological heat adaptation

To limit the development of physiological heat adaptation, the number of experiments was set to a maximum of three per week, but typically did not exceed two per week. Given that sustained elevations in core body temperature and skin temperature are required to elicit heat adaptation (Chen and Elizondo 1974) and that the fixed heart rate approach minimises elevations in core temperature (Fig. 5c), no relevant level of heat adaptation due to the testing is assumed. Moreover, the fixed heart rate method employed limits any potential effect of subjective adaptation to frequent heat exposure. Similarly, no impact of testing over a long period with different seasons is expected in the present experiment as prior research shows no significant seasonal effect on thermal or cardiovascular responses to work in the heat (Bain and Jay 2011), reducing the possibility of heat adaptation that could have impacted the results. However, since some participants completed many trials, we cannot completely dismiss the influence of mild heat adaptation over such a long experimental period. Due to potential thermoregulatory adaptations, individuals were not permitted to take part in any experimental procedures if they were heat acclimated or acclimatised (Garrett et al. 2011), e.g. by spending time in very hot climates or by participating in acclimatisation experiments.

### Experimental controls

Individual participants completed experimental sessions at the same time of day to minimise the effect of circadian rhythm on outcome variables (Waterhouse et al. 2004). Participants were asked to arrive hydrated before commencement of laboratory testing sessions and to refrain from caffeine on the day of each trial and alcohol and vigorous exercise 24 h prior to each trial.

### Anthropometry and submaximal exercise/fitness test (Visit 1)

Anthropometry was conducted with participants wearing shorts, T-shirt, and socks. Body mass was measured to the nearest gram using a high-precision digital scale (Metter Toledo kcc150, Metter Toledo, Leicester, UK), and stature was measured to the nearest 0.01 m using a wall-mounted stadiometer (Holtain, Crosswell, UK). Body composition was determined by bioelectrical impedance (Tanita MC-780MA, TANITA Corporation, Tokyo, Japan).

A gradient-based incremental submaximal exercise test was conducted in an environmental chamber regulated at 18°C, 40% rh and was performed on a treadmill (Mercury Medical, h**/**p**/**cosmos sports & medical Gmbh, Germany). This test consisted of a maximum of six, 3-min stages. During the test, the treadmill speed was fixed at 4.5 km·h^−1^, and the gradient was increased by 5% every 3 min until a steady-state heart rate of 85% age-predicted maximum was elicited. Expired air and heart rate were continuously monitored using an online gas analysis system (Quark CPET, COSMED, Albano Laziale, Rome) and short-range telemetry (Polar PE4000, Polar Electro, Kempele, Finland), respectively. The oxygen uptake and heart rate data collected during the submaximal treadmill test were extrapolated to estimate maximal oxygen consumption (*V̇*O_2max_) (American College of Sports Medicine 2013).

### Experimental trials

Upon arrival, participants inserted a rectal thermistor (VIAMED, Yorkshire, UK) to a depth of 10 cm past the anal sphincter. They then provided a urine sample for assessment of urine specific gravity. To ensure a state of euhydration, if urine specific gravity was > 1.020, participants were asked to drink ~500 mL water and to provide a second urine sample after 20 min (Armstrong et al. 1994). To monitor skin temperature (*T*_skin_), skin thermistors were placed on the belly of the *pectoralis major (T*_chest_*)*, *triceps (T*_arm_*)*, *rectus femoris (T*_thigh_*)*, and *gastrocnemius (T*_calf_*)*. The mean *T*_skin_ was then calculated based on the equation provided by Ramanathan (Ramanathan 1964). The value for *T*_skin_ was reported as the average score during a hot work trial.

$$ {T}_{\mathrm{skin}}=0.3\left({T}_{\mathrm{chest}}+{T}_{\mathrm{arm}}\right)+0.2\left({T}_{\mathrm{thigh}}+{T}_{\mathrm{calf}}\right)\ \left[{}^{\circ}\mathbf{C}\right] $$

Participants entered the environmental chamber wearing one of two ensembles. In the low-clothing coverage trials, participants donned underwear, standardised shorts, socks, and trainers. In the high-clothing coverage trials, the participants donned underwear, a standardised cotton t-shirt, and an appropriately sized, standardised full body protective coverall (65% polyester, 35% cotton). The intrinsic clothing insulations of the low- and high-clothing coverage ensembles were estimated as 0.04 and 0.133 m^−2^·K·W^−1^ (0.26 and 0.86 Clo), respectively, based on the reference tables provided in the international standard (ISO9920 2009). Using Eq. 31 in the standard, the evaporative resistance can be estimated as 0.007 and 0.024 m^−2^·kPa·W^−1^ for the low- and high-clothing coverage ensembles, respectively.

Various data acquisition systems were used to log skin and core temperature (Grant Squirrel SQ2020, Grant Instruments Ltd., Corby, UK), WBGT (Quest temp model 34), air temperature, relative humidity, and air velocity (Testo Ltd, model 435-2 Alton, Hampshire, UK) at 1-min intervals. Ratings of thermal comfort, rating of perceived exertion (RPE), and thermal sensation were taken every 5 min and reported as median of the work trial. Images of the scales used are shown in the online supplement (Figure S4). ‘The descriptors for each of the scales comply with international standards for measuring thermal comfort and sensation’ (ISO10551 2001). The comfort and sensation scales were modified to include intermediate numeric values to provide more choice to the participants regarding their precise comfort and thermal sensation level. The term ‘extremely uncomfortable’ was not included in our scale due to its limited practical use in our work.

Participants were removed from the climate chamber and the trial terminated if core temperature reached 39°C.

### Physical work simulation

One hour of treadmill-based walking was then commenced adhering to the following protocol. The treadmill was programmed to control workload to achieve the desired heart rate of 130 b·min^−1^. With this setting applied, the treadmill automatically manipulated the speed and grade to ensure heart rate was maintained at the predefined target. The treadmill speed and grade were never manually controlled by the researchers or participants. The treadmill elevation remained at 0% until the speed reached the threshold 6 km·h^−1^; thereafter, the treadmill regulated the elevation based on the difference between the actual and desired heart rate. The maximum test duration was set at 1 h, but exercise ceased if the treadmill speed reached zero, i.e. the participant having a heart rate of 130 b·min^−1^ at rest. In this scenario, participants were removed from the chamber and the trial did not continue.

### Calculation of percentage physical work capacity

The ‘minimum mechanics’ model was used to predict walking metabolism (kJ) (Ludlow and Weyand 2017). This equation was preferred over the widely used American College of Sports Medicine (ACSM) and Pandolf equations (Pandolf et al. 1977) due to its consistently stronger predictive accuracy over a wide range of speeds and grades (Ludlow and Weyand 2017). PWC was based on the total energy generated/expenditure (EE) above resting in each trial relative to that achieved in a cool reference condition, expressed as a percentage. The rate of work EE in kilojoules per minute (kJ·min^−1^) during each minute of work was calculated as below in Eq. 1, with the cumulative total used as final work EE (in kJ):

1 $$ Work\  EE={\sum}_{t=1}^{60}\left[0.32\cdotp G(t)+3.28+\left(1+0.19\cdotp G(t)\right)\cdotp \left(2.66\cdotp v{(t)}^2\right)\right]\cdotp \left(19.61+\frac{RQ(t)-0.707}{0.293}\cdotp 1.51\right)\ \left[ kJ\right] $$

where G(t) is the slope of the treadmill expressed in percent grade at time *t*, *v*(t) is velocity of walking expressed in meters per second, and RQ(t) is the respiratory quotient that was assumed to be 0.85 (Cramer and Jay 2019). The summation function (∑) denotes that the output of the equation is summed every 1 min (*t* = 1) until a stopping point of 60 min, accounting for the change in each variable over time. Part 1 of the equation (Ludlow and Weyand 2017) calculates the net volume of oxygen consumed (*V̇*O_2-net_, in mL·kg body mass^−1^·min^−1^) to fuel exercise, i.e. not including resting *V̇*O_2_. Part 2 of the equation converts the former into kJ.min^−1^. The cumulative EE for each trial was used to calculate total EE (in kJ) in each individual trial. The validity of the prediction equation for EE was tested against 365 expired air samples (5-min average) from a metabolic cart (Quark CPET, COSMED, Albano Laziale, Rome). Expired air measurements were taken at 3 time points (5–10, 30–35, and 50–55 min), and the kJ·min^−1^ subsequently compared with the prediction equation (Ludlow and Weyand 2017). See supplementary file for correlations and Bland-Altman analysis (Figure S5 and S6).

**Fig. 1:**
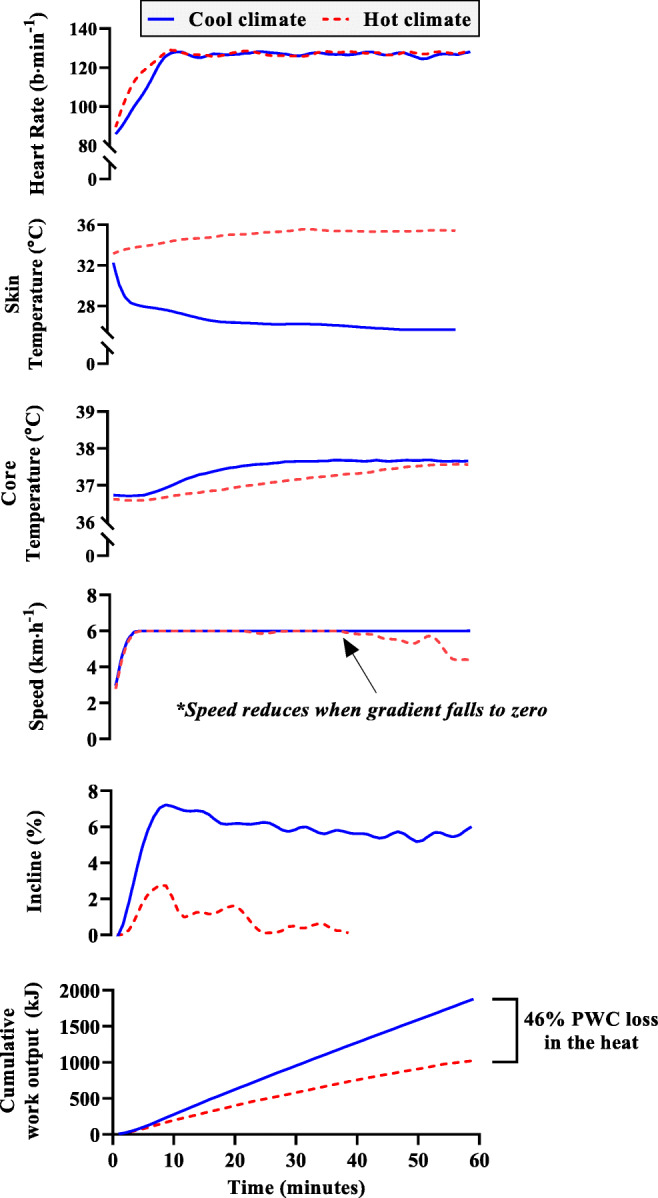
Fixed heart rate protocol measures how climatic heat impacts work capacity. Field studies demonstrate that individuals work at a similar heart rate despite changes in climatic heat due to adjustments in pacing. Although the heart rate profile is not different between conditions, heat stress causes a substantial increase in skin temperature, causing dramatic reductions in overall energy generated. Core (rectal) temperature responses in the early phase of the exposures are driven primarily by metabolic heat production (work rate). Changes to cumulative energy expenditure were used to calculate physical work capacity (PWC). Example data is shown for a representative participant for the reference condition (15°C, 50% humidity, solid blue line) and a hot condition (40°C, 20% humidity, dashed red line)

Percentage PWC in each hot trial was determined by expressing the total energy generated from metabolic processes (above resting) in the hour of work relative to that achieved in a reference cool condition (Eq. 2)

2 $$ \boldsymbol{Physical}\ \boldsymbol{Work}\ \boldsymbol{Capacity}=\left(\frac{Ho{t}_{kJ}}{Coo{l}_{kJ}}\right)\times 100\kern2em \left(\%\right) $$

where *Cool*_kJ_ is the total energy generated (kilojoules, kJ) above resting metabolism in the cool reference condition and *Hot*_kJ_ is the total energy generated above resting metabolism in each heat stress trial. Empirical models for the decrease in PWC under heat stress were then generated using data from 338 trials separated into low (181trials) and high (157 trials) clothing coverage. Figure 1 demonstrates how environmental heat stress changes skin temperature and consequently treadmill (physical work) parameters for a representative participant.

**Fig. 2:**
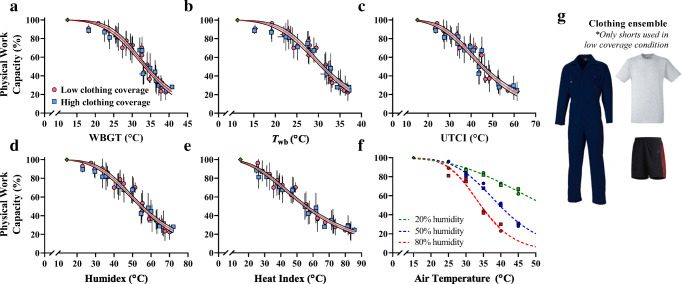
Models for the reduction in physical work capacity during heat stress. Models are presented against five heat stress indices (**a-e**) and for air temperature and relative humidity (**f**). The data used to form projections are taken as the average physical work capacity from each air temperature and humidity combination, pooling that of low- (pink circles) and high- (blue squares) clothing coverage trials (displayed in panel *g*). Model *f* was the highest performing overall, but model *e* (heat index) was the highest performing among different heat stress indices. Model analytics are available in Table 3

Figure 2 illustrates the two clothing ensembles worn in the low- and high-clothing coverage conditions. All trials were conducted in a still environment (~0.2 m·s^−1^), and scores for PWC used in the model were taken as the average for a group of participants in each climate condition. Low- and high-clothing coverage trials were completed in 22 climatic conditions, with an average of 7 trials per condition. The performance results for individual climates are presented in Table 2.

**Table 2:** Percentage physical work capacity across a broad range of air temperature and relative humidity combinations with high- and low-clothing coverage

Low-clothing coverage			High-clothing coverage		
*T*_a_	RH	PWC% ± SD	*T*_a_	RH	PWC% ± SD
25			25		
	20	91 ± 4		20	88 ± 3
	50	96 ± 1		--------------------	
	80	89 ± 11		80	81 ± 12
30			30		
	20	88 ± 11		20	87 ± 7
	50	84 ± 10		50	81 ± 11
	80	77 ± 11		80	75 ± 13
35			35		
	20	84 ± 9		20	82 ± 15
	35	70 ± 11		--------------------	
	50	73 ± 10		50	68 ± 14
	80	44 ± 12		80	42 ± 11
40			40		
	20	73 ± 10		20	71 ± 14
	40	70 ± 5		40	68 ± 13
	50	52 ± 14		50	50 ± 14
	60	36 ± 4		60	48 ± 16
	70	34 ± 13		70	35 ± 11
	80	23 ± 10		80	30 ± 11
45			45		
	20	62 ± 9		20	68 ± 15
	40	37 ± 15		40	45 ± 13
	50	28 ± 9		50	31 ± 10
	60	22 ± 8		60	25 ± 3
50			50		
	30	33 ± 5		30	29 *
	40	24 ± 3		40	28 *

*T*_*a*_ air temperature, *RH* relative humidity, *PWC%* percentage physical work capacity, *SD* standard deviation

^*^No standard deviation reported because *n* = 1

### Calculation of heat stress indices

#### Wet bulb globe temperature

The WBGT was measured empirically using a WBGT monitor (Quest temp model 34). The value used in the model was the average over the course of each work bout.

#### Aspirated (psychrometric) wet bulb temperature

Aspirated wet bulb temperature was calculated based on the formula provided by Bernard and Pourmoghani (1999):

3 $$ {T}_{wb}=0.376+5.79{P}_{\mathrm{a}}+\left(0.388-0.0465{\mathrm{P}}_a\right){T}_{\mathrm{db}}\kern0.5em \left[{}^{\circ}\mathrm{C}\right] $$

where *P*_a_ is the ambient water vapour pressure measured in kPa and *T*_db_ is the dry bulb temperature (air temperature). *P*_a_ was calculated by (Parsons 2010):

4 $$ {P}_{\mathrm{a}}=\left[{e}^{\left(18.956-\frac{4030.18}{T_{\mathrm{a}}+235}\right)}\right]\times \frac{Rh}{100}\kern0.5em \left[\mathrm{kPa}\right] $$

where *T*_a_ is the ambient temperature in °C and RH is the relative humidity (0–100).

#### Universal thermal climate index

The UTCI was determined using an excel calculator (www.climatechip.org/excel-wbgt-calculator), using the regression polynomial provided by the operational procedure of UTCI (Bröde et al. 2012). The input values used for the calculation were *T*_a_, RH, globe temperature, and air velocity. The UTCI macro was converted into VBA from the FORTRAN source code supplied at the UTCI site (www.utci.org/utci_doku.php).

#### Humidex

The humidex was calculated based on Masterton and Richardson (1979) and Rana et al. (2013):

5 $$ \mathrm{Humidex}={T}_{\mathrm{a}}+\frac{5}{9}\left(\left[6.112\times {10}^{\left(\frac{7.5{T}_{\mathrm{a}}}{237.7+{T}_{\mathrm{a}}}\right)}\times \frac{Rh}{100}\right]-10\right)\kern0.5em \left[{}^{\circ}\mathrm{C}\right] $$

#### Heat index

The heat index was calculated based on Rothfusz (1990):

6 $$ Heat\ Index=-42.379+2.04901523{T}_a+10.14333127 Rh-0.22475541{T}_a\bullet Rh-6.83783\times {10}^{-3}{T}_a^2-5.481717\times {10}^{-2}R{h}^2+1.22874\times {10}^{-3}{T}_a^2\bullet Rh+8.5282\times {10}^{-4}{T}_a\bullet R{h}^2-1.99\times {10}^{-6}{T}_a^2\bullet R{h}^2\left[{}^{\circ}F\right] $$

where *T*_a_ is in degrees Fahrenheit and RH is 0–100. The heat index in Fahrenheit (*HI*_F_) was converted to degrees Celsius by:

7 $$ \mathrm{Heat}\ \mathrm{Index}=\left(H{I}_{\mathrm{F}}-32\right)\times \frac{5}{9}\kern0.5em \left[{}^{\circ}\mathrm{C}\right]\kern0.75em $$

### Statistical analysis

All statistical models using heat stress indices were generated using GraphPad Prism version 8. Models incorporating air temperature and relative humidity were generated using nonlinear equation builder in IBM SPSS Statistics version 25. The reduction in PWC caused by heat was modelled using the collected data in relation to (i) commonly used heat stress indices and (ii) air temperature and humidity. Heat stress indices included in the analysis are the WBGT, aspirated (psychrometric) wet bulb temperature (*T*_wb_), universal thermal climate index (UTCI), humidex, and heat index (see methodology for calculations). Additional formulas are available in the online supplement if using apparent temperature, standard effective temperature, the Oxford index, perceived temperature, physiological equivalent temperature, and the modified physiological equivalent temperature (Tables S4, S5, and S6). Each index is used as a general climate strain index, and we acknowledge in Table 3 that some indices (i.e. heat index) do not account for solar radiation and wind speed in their calculation. Such parameters are being considered in ongoing works from our lab in which we assess their independent effects on PWC across the heat stress spectrum (Foster et al. 2020b; Smallcombe et al. 2019b). These indices were chosen due to their frequent application in occupational hygiene, climate modelling, biometeorology, and weather reports.

**Table 3:** Equations linking physical work capacity to the thermal climate

Heat stress metric	Range	Equation: PWC=	*R*^2^	RMSE	Index accounts for solar radiation?
*T*_a_ and humidity	*T*_a_ 15–50°C RH 20–80%	$\frac{100}{1+{\left[\frac{\left(-12.28 Ln\Big(\mathrm{RH}\right)+87.99}{T_{\mathrm{a}}}\right]}^{\left[-2.21 Ln\left(\mathrm{RH}\right)+2.63\right]}}$	.98	3.09	No
Heat index	14–85°C	$\frac{100}{1+{\left(\frac{55.47}{\mathrm{heat}\ \mathrm{index}}\right)}^{-2.90}}$	.97	4.10	No
Humidex	13–71°C	$\frac{100}{1+{\left(\frac{54.50}{\mathrm{humidex}}\right)}^{-4.10}}$	.96	4.86	No
*T*_wb_	10–39°C	$\frac{100}{1+{\left(\frac{30.98}{T_{\mathrm{wb}}}\right)}^{-5.90}}$	.95	5.60	No
UTCI	15–63°C	$\frac{100}{1+{\left(\frac{45.33}{\mathrm{UTCI}}\right)}^{-4.30}}$	.94	5.90	Yes
WBGT	12–40°C	$\frac{100}{1+{\left(\frac{33.63}{\mathrm{WBGT}}\right)}^{-6.33}}$	.94	5.94	Yes

*WBGT* wet bulb globe temperature, *T*_*wb*_ aspirated wet bulb temperature, *UTCI* universal thermal climate index, *T*_*a*_ air temperature, *RMSE* root-mean-square error, *Ln* natural logarithm

In line with the approach of Kjellstrom et al. (2018), we considered a sigmoidal model to be the most appropriate fit to our data. A sigmoidal model allows for logical upper and lower limits of 100 and 0% PWC, respectively, which can be enclosed in one equation without adding limiters.

The data was modelled according to the following formula in Eq. 8:

8 $$ \boldsymbol{Physical}\ \boldsymbol{Work}\ \boldsymbol{Capacity}\%=\frac{100}{1+{\left(\frac{PWC50}{x}\right)}^{HillSlope}} $$

where *PWC50* is the value of *x* that elicits 50% PWC and *HillSlope* defines the steepness of the curve. The function has a ‘top’ and ‘bottom’ plateau of 100 and 0%, respectively, but we stress that our models are only validated within the span of conditions tested (see Table 2), where PWC reached a minimum of 20% in our cohort of participants. In GraphPad Prism, the ‘top’ and ‘bottom’ parameters were fixed at 100 and 0, respectively. The *HillSlope and PWC50* parameters were calculated from the software to find the optimal fit to the data (producing the least variance).

The model equation template was adapted when incorporating *T*_a_ and humidity only, instead of using a heat stress index. Therefore, if using *T*_a_ as the value of *x*, the values of *PWC50* and *Hillslope* are replaced with functions/models that represent their change as a function of relative humidity (Eq. 9):

9 $$ \boldsymbol{Physical}\ \boldsymbol{Work}\ \boldsymbol{Capacity}\%=\frac{100}{1+{\left[\frac{\left(a\cdotp Ln(RH)+b\right)}{T_a}\right]}^{\left[c\cdotp Ln(RH)+d\right]}} $$

where *RH* is the relative humidity (data valid from 20 to 80%) and Ln(*RH)* denotes a transformation of *RH* to the natural logarithm; the *a*, *b*, *c*, and *d* parameters are empirically derived constants.

#### Parameter tuning

The models that replace ‘*PWC50*’ and ‘*Hillslope*’ were derived by the following methodology.

1. For three different relative humidity levels (20, 50, and 80%), independent sigmoidal models were produced, which describe the loss in PWC as a function of air temperature (Fig. 2f). All three humidity models produce different values for ‘*PWC50*’ and ‘*Hillslope*’ to fit the data.
2. Basic linear models were then produced, which show how each of these parameters changes as a function of relative humidity. For example, relative humidity is the *x* value, and *PWC50* is the *y* value in the equation. Repeating this step for *Hillslope*, two models have now been generated, which describe how *PWC50* and *Hillslope* change as a function of relative humidity.
3. The model framework from Eq. 9 was input into SPSS nonlinear equation builder. The initial ‘best guess’ estimates for the coefficients *a*, *b*, *c*, and *d* were derived from the models produced in step 2. Running the nonlinear equation in SPSS produces the model where PWC can be predicted from air temperature and relative humidity, as shown in Table 3.

## Results

Table 2 shows the change in PWC in each environmental condition, based on either low- or high-clothing coverage. Here, we show that PWC is strongly influenced by air temperature, humidity, and, to a mild extent in our study, the level of clothing coverage.

**Fig. 3:**
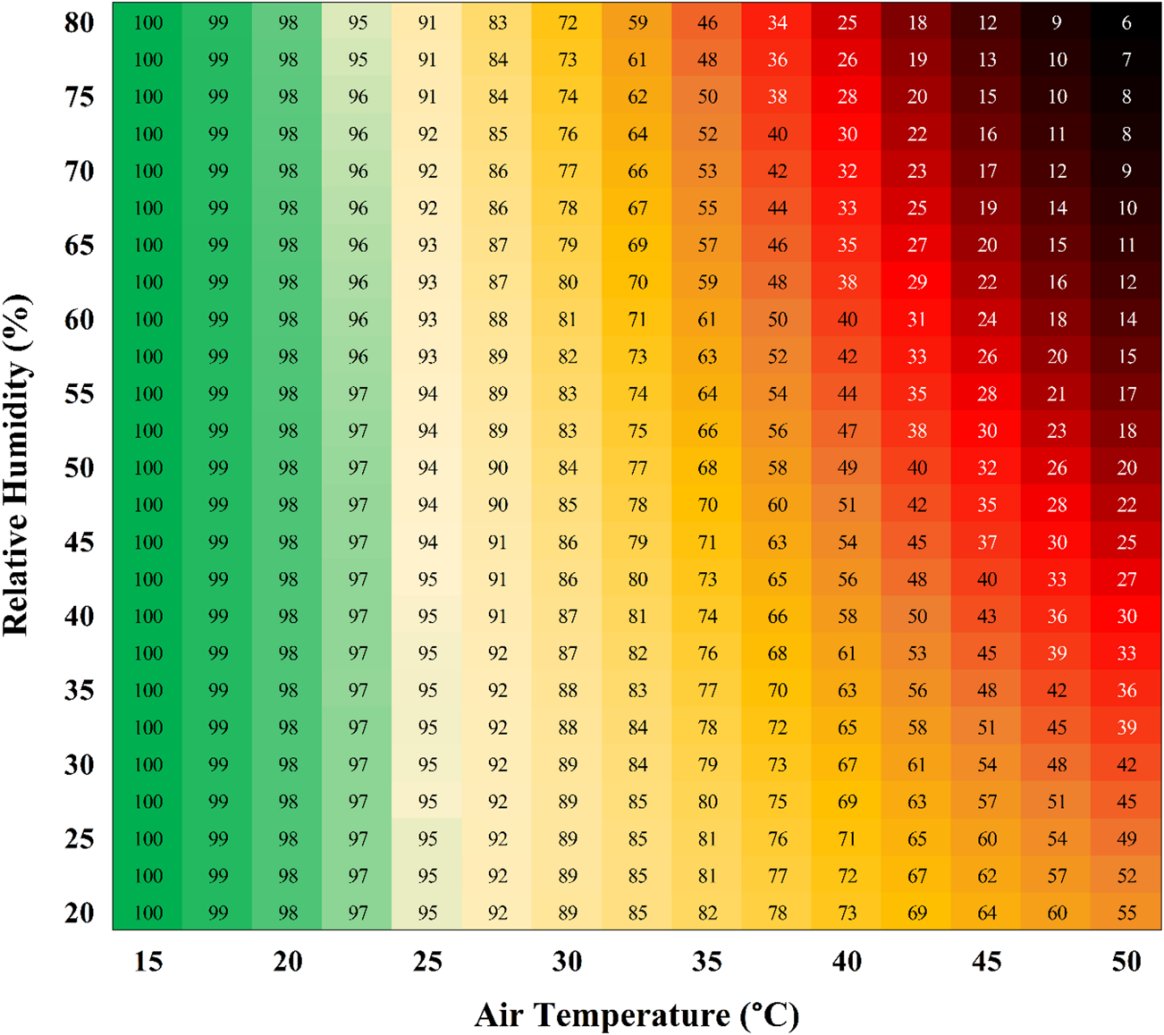
The change in physical work capacity percent as a function of air temperature and relative humidity. A graphical representation of the formula in Table 3 indicates the importance of humidity for the prediction of work capacity at a given air temperature. Values within the matrix indicate total physical work capacity as a percentage. Values are extrapolated where physical work capacity is < 25%

**Fig. 4:**
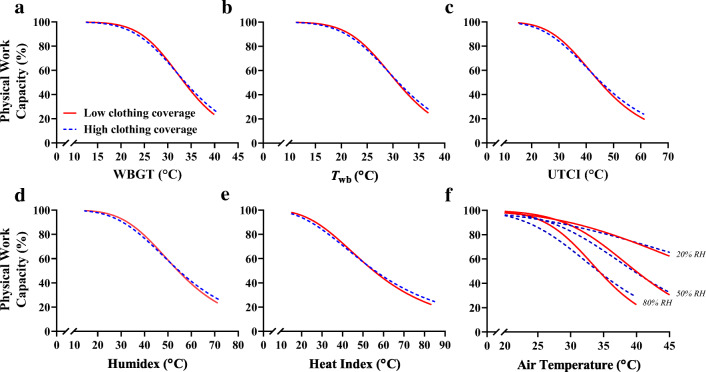
Clothing-specific models for physical work capacity. Models are presented based on low (red lines) or high (blue lines) levels of clothing coverage. Unlike the pooled data shown previously, the models here can be used for specific industries based on whether protective clothing is required. High-clothing coverage was detrimental in mild heat stress but offered some protective effect at more extreme heat. The thermal properties of each ensemble are described in the methods. Model analytics are available as supplementary material

**Fig. 5:**
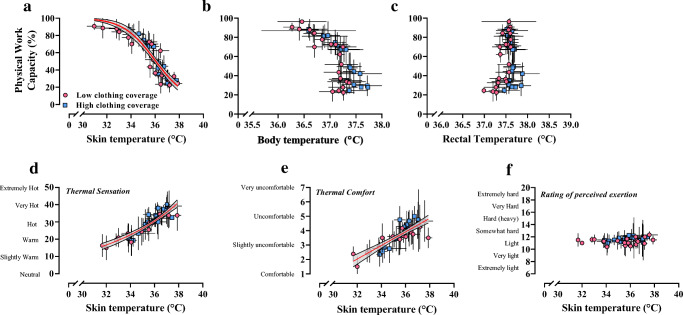
Performance and perceptual responses to heat are mediated by increased skin temperature. The change in physical work capacity (PWC) with heat (top panel) is predicted well by average skin temperature over the exposure (**a**), but not the average core (**b**) or the average mean body temperature (**c**). The data used to form projections are taken as the average physical work capacity from each air temperature and humidity combination, pooling that of low- (pink circles) and high- (blue squares) clothing coverage trials**.** Perceptual responses to heat can be predicted by skin temperature (**d** and **e**). There was no change in perceived exertion (**f**) with climatic stress, supporting the notion that perceived effort is primarily mediated by heart rate

Since the clothing types tested only had a limited impact on PWC, models were generated from the pooled dataset, as shown in Fig. 2a–e and separated for clothing (Fig. 4). The equations are displayed in Table 3 for the pooled clothing dataset. Equations for the separate low- and high-clothing coverage conditions shown in Fig. 4 are available in the supplementary material (Tables S1 and S2). Table 3 also acknowledges which equations are likely to be accurate in outdoor working conditions, with the added burden of thermal radiation from the sun, which increases heat strain and reduced physical performance independently of *T*_a_ and humidity (Hodder and Parsons 2007; Otani et al. 2016). Figure 3 shows the output from the *T*_a_ and humidity model as a matrix. The green area shows minimal reduction in PWC regardless of the *T*_a_ and humidity combination. The humidity has an increasingly strong impact on PWC at 25°C *T*_a_ and above. Figure 5 demonstrates the physiological manifestations underlying the observed reduction in PWC with heat. Whereas PWC was predicted strongly by the increase in skin temperature, rectal temperature and mean body temperature were not useful predictors. For those reasons, perceptual responses shown in Fig. 5 were expressed relative to the change in skin temperature only. Table 4 shows how PWC can be modelled based on skin temperature and how thermal sensation and thermal comfort can be predicted based on the skin temperature. Models are available in the supplementary material to predict skin temperature based on environmental heat stress indices.

**Table 4:** Equations for prediction of physical work capacity, thermal sensation, and thermal comfort due to changes in skin temperature during physical work

Outcome	Equation	*R*^2^	RMSE
Physical work capacity %	$\frac{100}{1+{\left[\frac{36.06}{T_{\mathrm{skin}}}\right]}^{-26.57}}$	.88	8.42
Thermal sensation	$0.13\cdotp {e}^{0.15{T}_{\mathrm{skin}}}$	.84	2.77
Thermal comfort	0.47*T*_skin_ − 13.06	.73	0.44

*T*_*skin*_ skin temperature in °C, *RMSE* root-mean-square error. Equations only valid for skin temperatures between 30 and 38°C

## Discussion

The aim of this study was to develop a series of empirical equations for the prediction of PWC in the heat. Using data for an average group of young males with heterogeneous fitness and body characteristics, we produced a series of equations where PWC can be predicted based on a series of heat stress assessment metrics, any combination of air temperature and relative humidity, or mean skin temperature. The workload employed is relevant to occupational scenarios, which require moderate to heavy physical work, such that we do not encourage the use of our findings for applications outside of this domain, i.e. maximal athletic performance. All equations took a nonlinear, sigmoidal form (Eqs. 8 and 9), which implies a flattening of PWC loss at the extreme heat levels (i.e. WBGT > 40°C). No field data exist that examines PWC at such extreme heat levels, and in line with previous suggestions, work output in these environments is more due to the heat capacity of the body, compared with the change in environmental heat load (Bröde et al. 2017; Kjellstrom et al. 2018); i.e. even in the most extreme conditions, a brief period of work *is* possible, assuming the worker can move to a cooler climate after the work is finished.

Figure 2 shows PWC based on several environmental assessment metrics. While Table 3 shows that the heat index formed models with the least residual variance, the WBGT and UTCI are able to account for solar radiation, so these models can be used for indoor and outdoor working scenarios. Our ongoing work aims to form correction factors so that indices that do not account for solar radiation can also be used to predict PWC outdoors (Foster et al. 2020b). A pooled model incorporating both clothing conditions is recommended for predicting PWC on a global scale, since it represents a generalised approximation that does not account for the subtle differences elicited by the clothing. The difference in parameter (*PWC50* and *Hillslope*) outcomes between conditions of high- and low-clothing coverage was consistent but minimal (Fig. 4). This is explained by the use of a reference condition in which the same clothing was worn, rather than comparing all to a minimally clothed condition (Fig. 5).

PWC as a function of air temperature and relative humidity is displayed as a contour plot in Fig. 3. Here, we show that *T*_a_ alone is a relatively poor predictor of PWC, which has implications for models that do not consider humidity in addition to temperature (Hsiang et al. 2017; Zivin and Neidell 2014). Regarding the clothing effect, it is shown in Fig. 4 that although the *PWC50* parameter (the value of *x* at 50% PWC) is similar between conditions, the *Hillslope* coefficient is smaller in the clothed condition, resulting in a shallower curve. The latter indicates a protective effect of high-clothing coverage in high temperatures (ambient temperature > skin temperature), but also a negative impact in milder conditions (skin temperature > ambient temperature). This notion is not surprising from a biophysical point of view and is supported by research examining the impact of clothing coverage in heat stressed humans (McLellan and Havenith 2016).

### Skin temperature predicts PWC and perceptual responses to heat stress

We additionally modelled the thermometric and perceptual responses across the full span of environmental conditions tested. At a fixed heart rate, which is a surrogate for self-paced physical workloads, Fig. 5a–c demonstrates that rising skin temperature mediates heat-induced reductions in PWC, whereas internal body temperature and mean body temperature (a weighted combination of skin temperature and core temperature) are not useful predictors. Although rising core temperature is the primary risk factor for heat stroke (Leon and Bouchama 2015), this event is rare in most occupations, especially if workers can self-pace (Miller et al. 2011). Rises in core temperature are primarily driven by metabolic workload (Cramer and Jay 2015), and since workload decreases in hot conditions due to self-pacing (Miller et al. 2011), core temperatures rarely reach 39°C in the field (Kalkowsky and Kampmann 2006; Miller et al. 2011) and in our dataset. In contrast, rising skin temperature is largely driven by the environment, where hot skin increases workers’ heart rate and perception of effort. We criticised the approach of Dunne et al. (2013), using the ACGIH work/rest recommendations to predict physical productivity, because the guidelines are designed to prevent core temperature exceeding 38°C and are thus highly conservative. Although our data indicate modest rises in core temperature, we emphasise that those values (as in Fig. 5c) are the average of a work bout, not the maximum. The core-to-skin temperature gradient equally shows good predictive value; however, further analysis showed that this is almost completely based on the skin temperature change.

The rating of perceived exertion (RPE) scale measures the participants’ subjective assessment of their physical effort (Borg 1982). The psychophysiological basis for the scale implies that RPE is strongly linked to cardiovascular strain, captured by heart rate, and thus, using the constant heart rate paradigm, RPE was expected to be constant. Figure 5f confirms that RPE did not change as a function of heat stress intensity, i.e. remains linked to heart rate in the heat, despite increases in thermal perception in hot climates (Fig. 5d and e). These results support recent commentary (Lloyd and Havenith 2019) and imply that reducing skin temperature should be the primary intervention to maintaining PWC and thermal perception in hot workplaces. Equations linking PWC to skin temperature, thermal sensation, and thermal comfort are displayed in Table 4.

### Comparison with previous models

**Fig. 6:**
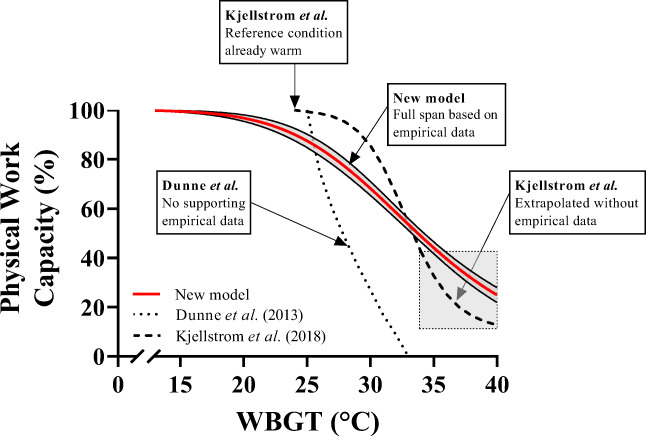
The relationship between PWC and WBGT for past models and the new model. Deriving relations based on work/rest ratios (dotted line, Dunne et al. (2013)) or from past field data (dashed line, Kjellstrom et al. 2018) underestimate the effect of moderate heat stress and overestimate the effects of extreme heat stress, resulting in unrealistically steep declines in PWC. The red line shows the new model, based on Fig. 2a, where we collected data up to 40°C WBGT. The new model is more sensitive to mild/moderate heat compared with previous work. The grey box indicates the area in which the Kjellstrom et al. (Kjellstrom et al. 2018) model is extrapolated beyond empirical data and an overestimation of the climate effect is present

It is useful to compare our model against WBGT-based models described in the introduction of our paper (Dunne et al. 2013; Kjellstrom et al. 2018). As shown in Fig. 6, a comparison is only available for WBGT since previous models were developed for this index only. Due to the incorporation of a cool reference condition and the high sensitivity of heart rate to changes in heat stress, our model better detects reductions in PWC in mild heat, compared to models developed by Dunne et al. (2013) and Kjellstrom et al. (2018), which only predict PWC reductions above 25°C WBGT. The high sensitivity of our model allows for a more accurate quantification of PWC with the more subtle climatic alterations observed in temperate climates, rather than the exclusive consideration of more severe conditions. Importantly, it is well established that 25°C WBGT already far exceeds optimal ambient conditions for human physical performance (Ely et al. 2007; Galloway and Maughan 1997; Taylor et al. 1963). In contrast, our reference condition (15°C;12°C WBGT) represents a more optimal environment for human physical work output (Taylor et al. 1963), allowing us to document PWC reductions in environments as mild as 18°C WBGT.

Although our model is more sensitive to low heat stress compared to both previous models, we also observed a shallower decline in PWC when WBGT surpassed 25°C compared with Dunne’s (2013) and Kjellstrom et al.’s (2018) models. PWC estimations from our model and that of Kjellstrom et al. (2018) produce similar values around 30–5°C WBGT, where the lines cross over. Due to Kjellstrom et al.’s choice to select 10% PWC as a lower limit, and the lack of data above 33°C WBGT, their predicted PWC values drop substantially below our data in this area. Our lowest PWC values observed were around 25–30% at 40°C WBGT and the curve suggests further drops above this level of heat stress. Such extreme WBGT values are very rarely encountered, but since workplace WBGT levels up to 35°C have been reported in various industries in India (Venugopal et al. 2015), workplace WBGTs ≥ 40°C are possible with future global warming.

### Limitations

There are several potential limitations of the current study that should be considered. The first is the use of mostly British participants, assumed to be unacclimatised to heat due to infrequent hot weather and minimal core temperature increases with our protocol, which are normally required for physiological adaptation (Fox et al. 1963). However, aerobic fitness is a major parameter that governs PWC in hot climates (Foster et al. 2020a), and thermoregulatory modelling indicates that those in a high state of acclimatisation (with normal fitness) exhibit a similar heat stress response to those of high fitness (Havenith 2001). The inclusion of participants with high fitness levels therefore does reflect part of the impact acclimatisation would have on the data. Although our model does not account for those who are simultaneously highly fit and acclimatised, these individuals are rare and account for 1–5% of the population average (Kaminsky et al. 2015).Second, only relatively young, healthy, adult males were recruited, raising the question about the older population and female workers. There is ample evidence however that both older and female workers, when healthy, respond similar to heat exposures as their younger counterparts, as long as their fitness levels and, to a lesser degree, their anthropometrics are considered (Cramer and Jay 2015; Havenith et al. 1995; Havenith and van Middendorp 1990; Notley et al. 2019). Hence, by incorporating a wide range of individual levels of fitness, the results of the present study should be representative of the general population, apart from very low fitness individuals, or those with underlying health conditions, who may respond differently. Thus, when translating to future impact assessment of global warming, our data represents the best-case scenario.

A third consideration is the choice for the type of work performed. Ideally, a range of work intensities and types of work would be studied, including lower working heart rates, upper body (manual work with hands arms mainly) only work, and combined upper and lower body work. Unfortunately, a study of the full range of specific activities in industry and their interaction with climate, while relevant, is not feasible, given the plethora of possible activities. In the paradigm used here, the work is performed mainly as lower body work. The main purpose of the work is to create the metabolic load as well as generate the associated heat in the body. In terms of the ratio of heat produced to metabolic rate, this would not be dissimilar for other work types. Further, the work level used does not induce excessive muscle fatigue in the tests; thus, different fatigue speeds for different work types would not be expected to have affected the outcomes.

A fourth consideration is that the equations presented are, at present, only valid for relatively low wind conditions and without solar radiation. Follow-up work from our group shows that the UTCI scale is the optimal climatic index when accounting for higher wind speeds and solar radiation (Foster et al. 2020b; Smallcombe et al. 2019b). The WBGT predicted PWC well during increased solar radiation, but does not account for the dynamic impact of wind; i.e. high wind can increase heat strain in hot dry environments (Morris et al. 2019; Smallcombe et al. 2019b). A fifth consideration is that the models presented in this paper (and their analytics) are developed from group means, and the reported accuracy is not representative of all individuals. Given the wide variation in fitness levels and body characteristics, our models represent a typical group of workers engaged in physical labour. Despite that, in our follow-up work, we modelled the impact of individual fitness on the PWC curves, explaining a large portion of the individual variation (Foster et al. 2021). It should also be noted that we considered using lower heart rates. Some sources suggest an average maximal heart rate over the full working day (rest+work periods average) of 110 b.min^−1^. When piloting this, we found already in the mid-range of heat stress that participants reached this at rest, so without activity. Thus, this was not a workable model. In real work, for such low work rates, workers would accept an increase of heart rate by heat exposure to around 130 b.min^−1^ as discussed, i.e. using their cardiovascular reserve. Thus, for very low workloads, the present model may be on the conservative side, as it does not include this reserve of increasing the HR to 130 from a lower work baseline.

A final potential limitation is the use of a 1-h work simulation, and not a full day work simulation. Our group is actively investigating the extent to which our model predicts PWC across a full working day, with preliminary results implying limited impact of work duration (1 versus 6 hourly work cycles in a day) on PWC until conditions are extreme (WBGT > 35°C) (Smallcombe et al. 2019a). It appears that our model predicts full day PWC within 5% for most relevant climates encountered on Earth and with existing climate conditions if water is available for rehydration ad libitum during rest periods.

## Conclusions

In summary, we provide new empirical models for changes to physical work capacity (PWC) under a wide variety of environmental conditions, which are based on a range of commonly used climate indices that can be used on the macro level to estimate the impact of heat on productivity and the cost of future climate change for physical work under different CO_2_ emission scenarios. In conjunction with weather forecasting, the model can also be used on the micro level to estimate day-to-day production losses within a given industry across the local climate range. To our knowledge, this is the first study to provide empirical estimations for PWC based on a large dataset over a wide range of climatic conditions using a wide variety of different heat indices, broadening the scope for future application.

## Supplementary Information

ESM 1 (DOCX 1524 kb)
