# Supplementary material for: An advanced empirical model for quantifying the impact of heat and climate change on human physical work capacity
Source: Int J Biometeorol. 2021 Mar 5;65(7):1215–29. doi: 10.1007/s00484-021-02105-0 (PMC8213606; doi:10.1007/s00484-021-02105-0)
Supplement: Supplementary file 1 — (DOCX 1524 kb) [file 484_2021_2105_MOESM1_ESM.docx]

**Supplementary material**

The supplementary file contains the following items:

1. Equations for physical work capacity (PWC) separated based on low and high clothing coverage.
2. Equations and figures for predicting skin temperature (Tsk) based on the different heat stress indices shown in the main paper.
3. Equations and figures for predicting PWC using selected heat stress indices commonly used but not presented in the main text.
4. Images of the perceptual scales used.
5. Measured vs predicted energy expenditure data

**Equations for physical work capacity assuming a specific clothing condition**

| **Table S1. New equations linking physical work capacity to the thermal climate with low clothing coverage** | | | | | |
| --- | --- | --- | --- | --- | --- |
| **Heat Stress**  **Metric** | **Range** | **Equation: PWC=** | **R^2^** | **RMSE** | **Accounts for solar radiation?** |
|  |  | ***Low clothing coverage conditions*** |  |  |  |
| *T*_a_ and humidity | *T*_a_  15-50°C  RH  20-80% | $\frac{100}{1+\left[ \frac{\left( -12.28Ln(\mathrm{RH} \right)+87.99}{T_{a}} \right]^{\left( -2.21Ln(RH) + 2.63 \right)}}$ | .99 | 2.01 | No |
| Heat Index | 14-85°C | $\frac{100}{1+\left[ \frac{54.28}{Heat Index} \right]^{-4.37}}$ | .98 | 3.84 | No |
| Humidex | 13-71°C | $\frac{100}{1+\left[ \frac{54.59}{\mathrm{Humidex}} \right]^{-4.55}}$ | .97 | 4.81 | No |
| UTCI | 15-63°C | $\frac{100}{1+\left[ \frac{45.08}{\mathrm{UTCI}} \right]^{-4.78}}$ | .96 | 5.58 | Yes |
| WBGT | 12-40°C | $\frac{100}{1+\left[ \frac{33.51}{\mathrm{WBGT}} \right]^{-6.76}}$ | .95 | 5.96 | Yes |
| *T*_wb_ | 10-39°C | $\frac{100}{1+\left[ \frac{30.87}{T_{\mathrm{wb}}} \right]^{-6.24}}$ | .95 | 6.15 | No |
| WBGT, wet-bulb globe temperature; *T*_wb_, aspirated wet-bulb temperature; UTCI, Universal Thermal Climate Index; *T*_a_, air temperature; RMSE, root-mean squared error | | | | | |

| **Table S2. New equations linking physical work capacity to the thermal climate with high clothing coverage** | | | | | |
| --- | --- | --- | --- | --- | --- |
| **Heat Stress**  **Metric** | **Range** | **Equation: PWC=** | **R^2^** | **RMSE** | **Accounts for solar radiation?** |
|  |  | ***High clothing coverage conditions*** |  |  |  |
| *T*_a_ and humidity | *T*_a_  15-50°C  RH  20-80% | $\frac{100}{1+\left[ \frac{\left( -12.28Ln(\mathrm{RH} \right)+87.99}{T_{a}} \right]^{\left( -2.21Ln(RH) + 2.63 \right)}}$ | .98 | 3.09 | No |
| Heat Index | 14-85°C | $\frac{100}{1+\left[ \frac{55.79}{Heat Index} \right]^{-2.68}}$ | .97 | 4.14 | No |
| Humidex | 13-71°C | $\frac{100}{1+\left[ \frac{54.71}{\mathrm{Humidex}} \right]^{-3.81}}$ | .96 | 4.81 | No |
| *T*_wb_ | 10-39°C | $\frac{100}{1+\left[ \frac{31.10}{T_{\mathrm{wb}}} \right]^{-5.54}}$ | .96 | 4.87 | No |
| WBGT | 12-40°C | $\frac{100}{1+\left[ \frac{33.76}{\mathrm{WBGT}} \right]^{-5.87}}$ | .94 | 5.84 | Yes |
| UTCI | 15-63°C | $\frac{100}{1+\left[ \frac{45.59}{\mathrm{UTCI}} \right]^{-3.98}}$ | .93 | 6.14 | Yes |
| WBGT, wet-bulb globe temperature; *T*_wb_, aspirated wet-bulb temperature; UTCI, Universal Thermal Climate Index; *T*_a_, air temperature; RMSE, root-mean squared error | | | | | |

**Equations for skin temperature if using a heat stress index as the predictor variable. Data plotted below in Figure S1.**

| **Table S3. New equations linking skin temperature to the thermal climate.** | | | | |
| --- | --- | --- | --- | --- |
| **Heat Stress**  **Metric** | **Equation: Tsk=** | **R^2^** | **RMSE** | **Accounts for solar radiation?** |
|  | ***Pooled clothing coverage conditions*** |  |  |  |
| WBGT | $0.264\mathrm{WBGT}+27.07$ | .90 | 0.52 | Yes |
| UTCI | $\left( 16.33-38.36 \right)e^{\left( -0.05097UTCI \right)}+38.36$ | .91 | 0.53 | Yes |
| *T*_a_ and humidity | $[\left( 0.027Ln\left( Rh \right)\cdot0.125 \right)\cdot Ta]+26.99$ | .89 | 0.60 | No |
| Heat Index | $\left( 18.28-37.33 \right)e^{\left( -0.05282HeatIndex \right)}+37.33$ | .88 | 0.60 | No |
| Humidex | $\left( 26.30-40.63 \right)e^{\left( -0.02094Humidex \right)}+40.63$ | .88 | 0.60 | No |
| *T*_wb_ | $\left( 26.46-40.20 \right)e^{\left( -0.03944Humidex \right)}+40.20$ | .75 | 0.86 | No |
| WBGT, wet-bulb globe temperature; *T*_wb_, aspirated wet-bulb temperature; UTCI, Universal Thermal Climate Index; *T*_a_, air temperature; RMSE, root-mean squared error | | | | |


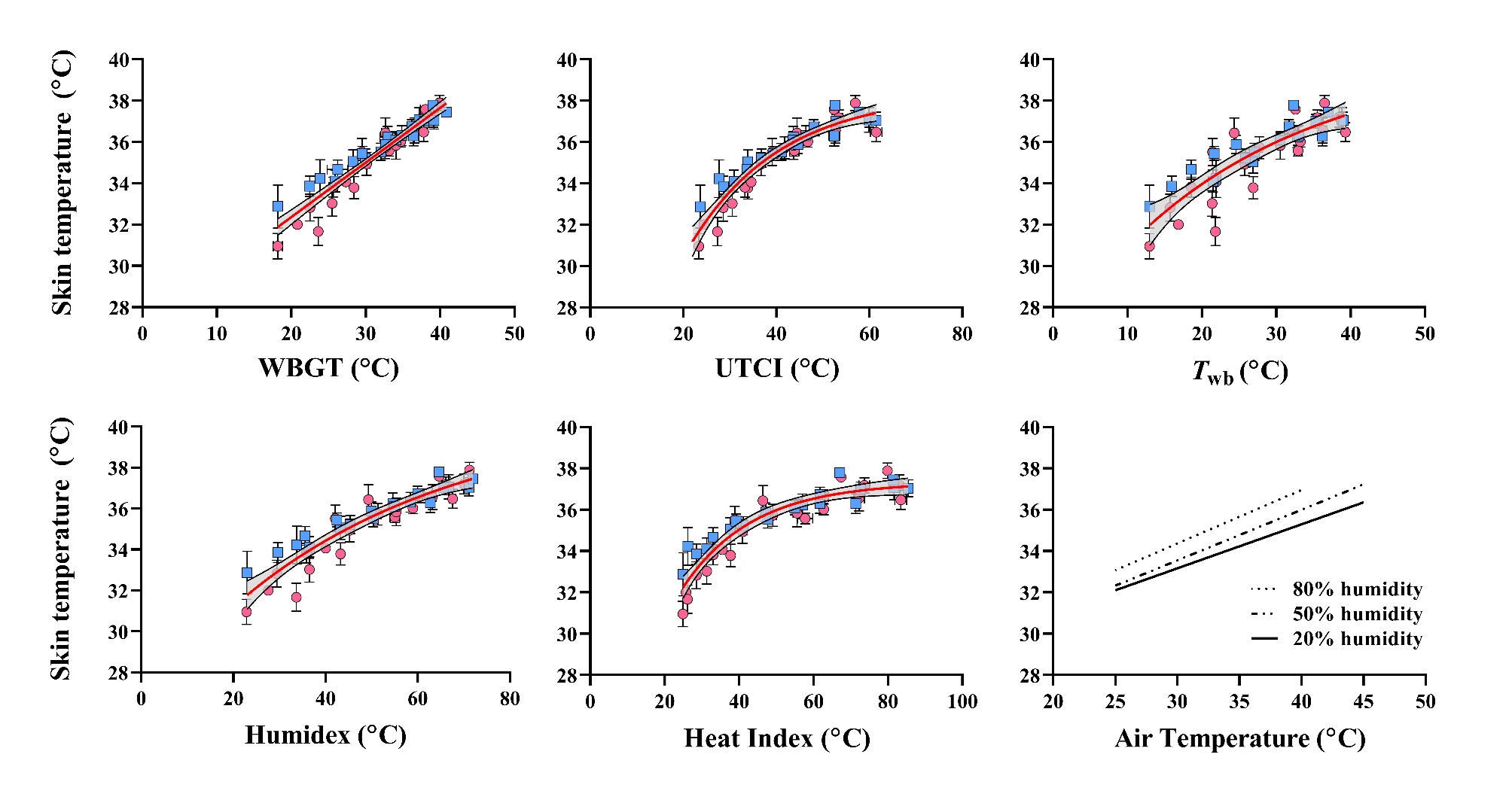


**Figure S1. Models for the prediction of mean skin temperature across different thermal climates and heat indices.** During occupational work, our paper indicates that skin temperature is the primary predictor of physical work capacity and perceptual responses to heat. Skin temperature can follow a linear or nonlinear (exponential decay) pattern which seems to be dependent on the unit spacing between heat stress intensities. The more compressed nature of the WBGT units result in a linear skin temperature rise, where alternate indices with larger unit spacing results in a nonlinear rise. Table S3 provides equations for each metric.

**Equations for physical work capacity for different heat stress indices**

Calculation of different indices

Physiological equivalent temperature (Höppe, 1999), modified physiological equivalent temperature (Chen and Matzarakis, 2018), Standard effective temperature (Gonzalez et al., 1974), and Perceived temperature (Gabriel, 1991) were calculated using RayMan software version 3.1 (Matzarakis et al., 2007, 2010).

The Oxford Index was calculated according to Lind and Hellon (Lind and Hellon, 1957):

$$Oxford Index \left( ^{\circ}C \right)=0.85T_{\mathrm{wb}}+0.15T_{a}$$

Where *T*_wb_ is aspirated (psychrometric) wet-bulb temperature (calculation shown in main text), and *T*_a_ is air temperature, both in degrees Celsius.

Apparent Temperature (Steadman, 1984) was calculated using the shaded version from the Australian Bureau of Meteorology (<http://www.bom.gov.au/info/thermal_stress/>). Use the alternative equation provided on the webpage if including solar radiation.

$$Apparent Temperature \left( ^{\circ}C \right)=T_{a}+0.33\cdot vp-0.7\cdot v-4.0$$

Where *T*_a_ is air temperature in Celsius, *vp* is ambient water vapour pressure in hPa, and *v* is air velocity in m/s (at 10m height). The ground level *v* was multiplied by 1.5 to find the approximate value at 10m height.

| **Table S4. Equations linking physical work capacity to the thermal. Indices are ranked based on their predictive power.** | | | | | |
| --- | --- | --- | --- | --- | --- |
| **Heat Stress**  **Metric** | **Range**  **(°C)** | **Equation: PWC=** | **R^2^** | **RMSE** | **Index accounts for solar radiation?** |
| Oxford Index | 11-39 | $\frac{100}{1+\left( \frac{31.55}{Oxford Index} \right)^{-4.75}}$ | 0.96 | 5.29 | No |
| Standard Effective Temperature | 18-47 | $\frac{100}{1+\left( \frac{40.25}{\mathrm{SET}} \right)^{-6.83}}$ | 0.95 | 5.85 | Yes |
| Perceived Temperature | 14-51 | $\frac{100}{1+\left( \frac{43.38}{\mathrm{PT}} \right)^{-4.01}}$ | 0.94 | 6.02 | No |
| Apparent Temperature | 14-62 | $\frac{100}{1+\left( \frac{47.48}{\mathrm{AT}} \right)^{-4.60}}$ | 0.94 | 6.15 | Yes |
| mPET | 16-49 | $\frac{100}{1+\left( \frac{40.40}{\mathrm{mPET}} \right)^{-5.29}}$ | 0.87 | 8.21 | Yes |
| Water Vapour Pressure | 0.6-5.9 | $\frac{100}{1+\left( \frac{3.65}{vp} \right)^{-2.09}}$ | 0.82 | 10.65 | No |
| *PET | 14-51 | $\frac{100}{1+\left( \frac{40.98}{\mathrm{PET}} \right)^{-4.51}}$ | 0.73 | 12.95 | Yes |
| *Air Temperature | 15-50 | $\frac{100}{1+\left( \frac{40.32}{T_{a}} \right)^{-4.70}}$ | 0.72 | 13.22 | No |
| mPET; modified physiologically equivalent temperature; PET, Physiological equivalent temperature. *We advise against using these metrics to calculate PWC due to poor predictive accuracy (see Figure S2). | | | | | |

| **Table S5. New equations linking physical work capacity to the thermal climate with low clothing coverage. Indices are ranked based on their predictive power.** | | | | | |
| --- | --- | --- | --- | --- | --- |
| **Heat Stress**  **Metric** | **Range** | **Equation: PWC=** | **R^2^** | **RMSE** | **Accounts for solar radiation?** |
|  |  | ***Low clothing coverage conditions*** |  |  |  |
| Standard Effective Temperature | 18-47 | $\frac{100}{1+\left( \frac{40.12}{\mathrm{SET}} \right)^{-7.36}}$ | 0.96 | 5.29 | Yes |
| Oxford Index | 11-39 | $\frac{100}{1+\left( \frac{31.42}{Oxford Index} \right)^{-5.03}}$ | 0.96 | 5.56 | No |
| Perceived Temperature | 14-51 | $\frac{100}{1+\left( \frac{43.17}{\mathrm{PT}} \right)^{-4.30}}$ | 0.95 | 5.89 | No |
| Apparent Temperature | 14-62 | $\frac{100}{1+\left( \frac{47.68}{\mathrm{AT}} \right)^{-4.93}}$ | 0.95 | 5.92 | Yes |
| mPET | 16-49 | $\frac{100}{1+\left( \frac{40.20}{\mathrm{mPET}} \right)^{-5.74}}$ | 0.89 | 8.06 | Yes |
| Water Vapour Pressure (kPa) | 0.6-5.9 | $\frac{100}{1+\left( \frac{3.65}{vp} \right)^{-2.27}}$ | 0.82 | 11.29 | No |
| *PET | 14-51 | $\frac{100}{1+\left( \frac{40.80}{\mathrm{PET}} \right)^{-4.92}}$ | 0.75 | 13.22 | Yes |
| *Air Temperature | 15-50 | $\frac{100}{1+\left( \frac{39.98}{T_{a}} \right)^{-5.13}}$ | 0.74 | 13.48 | No |
| mPET; modified physiologically equivalent temperature; PET, Physiological equivalent temperature. *We advise against using these metrics to calculate PWC due to poor predictive accuracy (see Figure S2). | | | | | |

| **Table S6. New equations linking physical work capacity to the thermal climate high clothing coverage. Indices are ranked based on their predictive power.** | | | | | |
| --- | --- | --- | --- | --- | --- |
| **Heat Stress**  **Metric** | **Range** | **Equation: PWC=** | **R^2^** | **RMSE** | **Accounts for solar radiation?** |
|  |  | ***High clothing coverage conditions*** |  |  |  |
| Oxford Index | 11-39 | $\frac{100}{1+\left( \frac{31.68}{Oxford Index} \right)^{-4.45}}$ | 0.96 | 4.89 | No |
| Perceived Temperature | 14-51 | $\frac{100}{1+\left( \frac{43.59}{\mathrm{PT}} \right)^{-3.69}}$ | 0.94 | 6.04 | No |
| Standard Effective Temperature | 18-47 | $\frac{100}{1+\left( \frac{40.38}{\mathrm{SET}} \right)^{-6.26}}$ | 0.93 | 6.27 | Yes |
| Apparent Temperature | 14-62 | $\frac{100}{1+\left( \frac{40.38}{\mathrm{AT}} \right)^{-6.26}}$ | 0.93 | 6.27 | Yes |
| mPET | 16-49 | $\frac{100}{1+\left( \frac{40.61}{\mathrm{mPET}} \right)^{-4.81}}$ | 0.86 | 8.39 | Yes |
| Water Vapour Pressure (kPa) | 0.6-5.9 | $\frac{100}{1+\left( \frac{3.65}{vp} \right)^{-1.89}}$ | 0.83 | 10.03 | No |
| *PET | 14-51 | $\frac{100}{1+\left( \frac{41.13}{\mathrm{PET}} \right)^{-4.07}}$ | 0.71 | 12.82 | Yes |
| *Air Temperature | 15-50 | $\frac{100}{1+\left( \frac{40.47}{T_{a}} \right)^{-4.22}}$ | 0.70 | 13.19 | No |
| mPET; modified physiologically equivalent temperature; PET, Physiological equivalent temperature. *We advise against using these metrics to calculate PWC due to poor predictive accuracy (see Figure S2). | | | | | |

**Figure S2. Models for the reduction in physical work capacity during heat stress.** Models for predictive PWC are presented based on selected heat stress indices. The data used to form projections are taken as the average physical work capacity from each air temperature and humidity combination, pooling that of low (pink circles) and high (blue squares) clothing coverage trials. Model analytics are available in Table 2.

**Figure S3. Clothing specific models for physical work capacity.** Models are presented based on low (red lines) or high (blue lines) levels of clothing coverage. Unlike the pooled data shown previously, the models here can be used for specific industries based on whether protective clothing is required. Model analytics are shown for low and hig coverage in Table S5 and S6, respectively.

**Perceptual Scales**


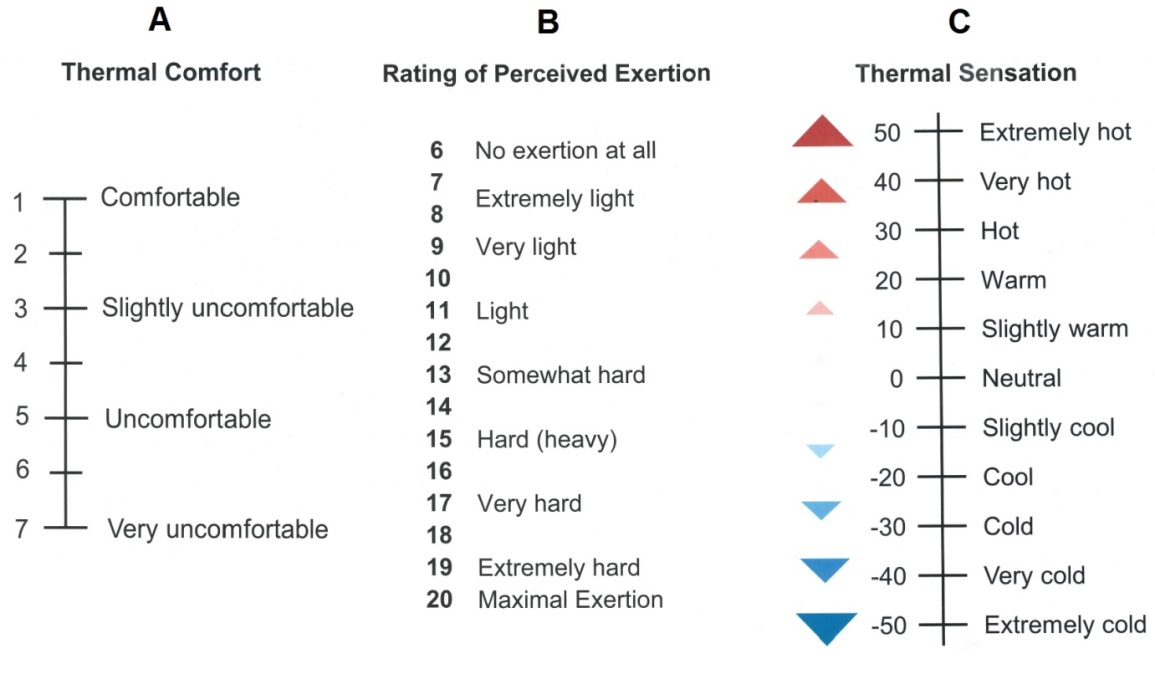


**Figure S4. Perceptual scales used for the assessment of thermal comfort (A), Rating of Perceived Exertion (B), and thermal sensation (C).**

**Measured vs predicted energy expenditure**

Figure S5 shows the measured vs predicted energy expenditure (kJ/min) at three distinct time points. Each data point is the average of either 5-10, 30-35, or 50-55 minutes. Despite the non-steady state nature of the fixed heart rate protocol, the equation (Ludlow and Weyand, 2017) provided a strong prediction of energy expenditure when compared with that collected from a metabolic cart (Quark CPET, COSMED, Albano Laziale, Rome). Figure S6 shows the data when analysed via a Bland Altman method (Altman and Bland, 1983).

**Figure S5.** Correlation between measured energy expenditure and that predicted from speed and grade (Ludlow and Weyand, 2017). There existed a strong correlation for the pooled data and for each timepoint. The solid black line is the line of identity.

**Figure S6.** Bland Altman plot showing the systemic differences between the measured and predicted energy expenditures. The dashed lines show the lower and upper limits of agreement. The dotted line shows the mean difference (bias).

**References**

Altman, D. G., and Bland, J. M. (1983). Measurement in Medicine: The Analysis of Method Comparison Studies. *Stat.* doi:10.2307/2987937.

Chen, Y. C., and Matzarakis, A. (2018). Modified physiologically equivalent temperature—basics and applications for western European climate. *Theor. Appl. Climatol.* 132, 1275–1289. doi:10.1007/s00704-017-2158-x.

Gabriel, W. (1991). Methodik zur räumlichen Bewertung der thermischen Komponente im Bioklima des Menschen - Fortgeschriebenes Klima-Michel-Modell. *Ann. Georgr.* 100, 492–492.

Gonzalez, R. R., Nishi, Y., and Gagge, A. P. (1974). Experimental evaluation of standard effective temperature a new biometeorological index of man’s thermal discomfort. *Int. J. Biometeorol.* 18, 1–15. doi:10.1007/BF01450660.

Höppe, P. (1999). The physiological equivalent temperature - A universal index for the biometeorological assessment of the thermal environment. *Int. J. Biometeorol.* 43, 71–75. doi:10.1007/s004840050118.

Lind, A. R., and Hellon, R. F. (1957). Assessment of physiological severity of hot climates. *J. Appl. Physiol.* 11, 35–40. doi:10.1152/jappl.1957.11.1.35.

Ludlow, L. W., and Weyand, P. G. (2017). Walking economy is predictably determined by speed, grade, and gravitational load. *J. Appl. Physiol.* 123, 1288–1302. doi:10.1152/japplphysiol.00504.2017.

Matzarakis, A., Rutz, F., and Mayer, H. (2007). Modelling radiation fluxes in simple and complex environments - Application of the RayMan model. *Int. J. Biometeorol.* 51, 323–334. doi:10.1007/s00484-006-0061-8.

Matzarakis, A., Rutz, F., and Mayer, H. (2010). Modelling radiation fluxes in simple and complex environments: Basics of the RayMan model. *Int. J. Biometeorol.* 54, 131–139. doi:10.1007/s00484-009-0261-0.

Steadman, R. G. (1984). A universal scale of apparent temperature. *J. Clim. Appl. Meteorol.* 23, 1674–1687. doi:10.1175/1520-0450(1984)023<1674:AUSOAT>2.0.CO;2.
